# Laser-Ablative Synthesis of Isotope-Enriched Samarium Oxide Nanoparticles for Nuclear Nanomedicine

**DOI:** 10.3390/nano10010069

**Published:** 2019-12-28

**Authors:** Elena Popova-Kuznetsova, Gleb Tikhonowski, Anton A. Popov, Vladimir Duflot, Sergey Deyev, Sergey Klimentov, Irina Zavestovskaya, Paras N. Prasad, Andrei V. Kabashin

**Affiliations:** 1 Bionanophotonic Lab., Institute of Engineering Physics for Biomedicine (PhysBio), National Nuclear Research University MEPHI, Moscow 115409, Russia; EAPopovaKuznetsova@mephi.ru (E.P.-K.); gtikhonowski@gmail.com (G.T.); deyev@ibch.ru (S.D.); SMKlimentov@mephi.ru (S.K.); INZavestovskaya@mephi.ru (I.Z.); 2 Karpov Institute of Physical Chemistry, NIFKhI, Obninsk 249033, Kaluga region, Russia; duflot@mail.ru; 3 Shemyakin–Ovchinnikov Institute of Bioorganic Chemistry, Russian Academy of Sciences, Moscow 117997, Russia; 4 Lebedev Physical Institute of the Russian Academy Sciences, Moscow 119991, Russia; 5 Department of Chemistry and Institute for Lasers, Photonics, and Biophotonics, University at Buffalo, The State University of New York, Buffalo, NY 14260, USA; 6 LP3, Aix Marseille University, CNRS, 13288 Marseille, France

**Keywords:** nuclear nanomedicine, pulsed laser ablation in liquids, samarium (Sm) oxide nanoparticles, femtosecond laser ablation and fragmentation

## Abstract

Nuclear nanomedicine is an emerging field, which utilizes nanoformulations of nuclear agents to increase their local concentration at targeted sites for a more effective nuclear therapy at a considerably reduced radiation dosage. This field needs the development of methods for controlled fabrication of nuclear agents carrying nanoparticles with low polydispersity and with high colloidal stability in aqueous dispersions. In this paper, we apply methods of femtosecond (fs) laser ablation in deionized water to fabricate stable aqueous dispersion of ^152^Sm-enriched samarium oxide nanoparticles (NPs), which can capture neutrons to become ^153^Sm beta-emitters for nuclear therapy. We show that direct ablation of a ^152^Sm-enriched samarium oxide target leads to widely size- and shape-dispersed populations of NPs with low colloidal stability. However, by applying a second fs laser fragmentation step to the dispersion of initially formed colloids, we achieve full homogenization of NPs size characteristics, while keeping the same composition. We also demonstrate the possibility for wide-range tuning of the mean size of Sm-based NPs by varying laser energy during the ablation or fragmentation step. The final product presents dispersed solutions of samarium oxide NPs with relatively narrow size distribution, having spherical shape, a controlled mean size between 7 and 70 nm and high colloidal stability. The formed NPs can also be of importance for catalytic and biomedical applications.

## 1. Introduction

Being a member of the lanthanide family, Samarium (Sm) exhibits a series of physicochemical properties, which favor its applications in many areas, including the development of strong magnets [1], control rods of nuclear reactors [2], efficient catalysts [3,4], and agents for biomedicine [5,6,7]. An important application of the ^152^Sm isotope is in nuclear medicine. This isotope captures neutrons by a nuclear reaction, ^152^Sm (n, γ) ^153^Sm, to produce the short-lived samarium-based radio isotope ^153^Sm, which is known as one of most promising beta emitters for the treatment of malignant tumors, including lung, prostate and breast cancers [6,7].

Nanoscale formulations of Sm are of particular importance, as they can offer a series of additional advantages, including a large surface area for catalysis [4] and other attractive properties for biomedical applications [8]. Nanoformulations of ^152^Sm-enriched samarium compounds are of great value for advancing nuclear nanomedicine as it can provide a high local concentration of the radioisotope. Prospects of Sm-based nanoformulations in this diverse range of applications critically depend on the surface conditioning and purity of nanomaterials, but currently-existing routes for the synthesis of Sm nanostructures do not always meet these requirements. Indeed, chemical methods for the preparation of Sm nanoparticles (NPs) [4,9] typically involve hazardous precursors, which could cause residual contamination leading to toxicity issues. Alternative synthesis techniques based on cluster beam deposition [10] and mechanical milling [11] are capable of producing NPs in the dry state, but their subsequent dispersion and stabilization in solutions is problematic due to the high surface energy of nanomaterials. Therefore, a facile and scalable technique for the production of Sm-based NPs is still required. Making possible fast production of bare (uncovered) NPs in colloidal state with almost any composition, pulsed laser ablation in liquids [12,13,14,15,16,17] (PLAL) provides one of best alternatives to satisfy the above-stated demands. As an example, we recently showed that the technique of femtosecond laser ablation in liquids can be used for the fabrication of a variety of ultrapure, biologically-friendly nanomaterials, including gold NPs [16,18,19,20,21,22], titanium nitride (TiN) NPs [23], silicon NPs [24,25,26,27] and organic polymer NPs [28]. In many cases, such nanoparticles can provide superior properties for catalytic [20], energy and biomedical [29,30] applications, compared to nanomaterials synthesized by conventional chemical methods, and other methods.

Here, we report on laser-ablative synthesis and structural characterization of pure spherical and size-tunable (7–70 nm) ^152^Sm-enriched samarium oxide NPs which can be suitable for nuclear nanomedicine tasks after activation in a nuclear reactor and appropriate biofunctionalization to minimize immune response and target tumors. We believe that such NPs can also be promising for catalytic and biomedical tasks.

## 2. Materials and Methods

### 2.1. Sm Oxide Powder and Preparation of Target for Ablation

**Figure 1:**
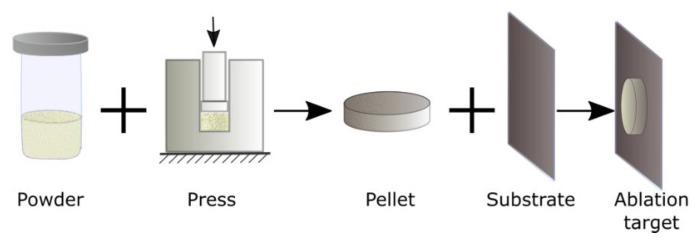
Schematic representation of ablation target preparation. The initial Sm oxide micro powder was pressed into a cylindrical pellet, which was then glued to a silicon wafer to form the ablation target.

We used samarium oxide micropowder enriched with ^152^Sm isotope (97.78–98.70%, purity 99.80%) purchased from Elektrokhimpribor Combine (Lesnoy, Sverdlovsk Region, Russia). The average grain size of the initial micro powder was 0.5–2 µm. The target was prepared as follows: Five milligrams of the powder was pressed into a cylindrical pellet having 5 mm diameter and 150 µm thickness. The pellet was then glued to a silicon substrate using epoxy glue to form the ablation target. Since the glue was used only on the back side of the pellet in order to fix it on the silicon substrate, it was not affected by laser radiation during our experiments. The target preparation process is shown schematically in Figure 1.

### 2.2. Synthesis of Nanoparticles

**Figure 2:**
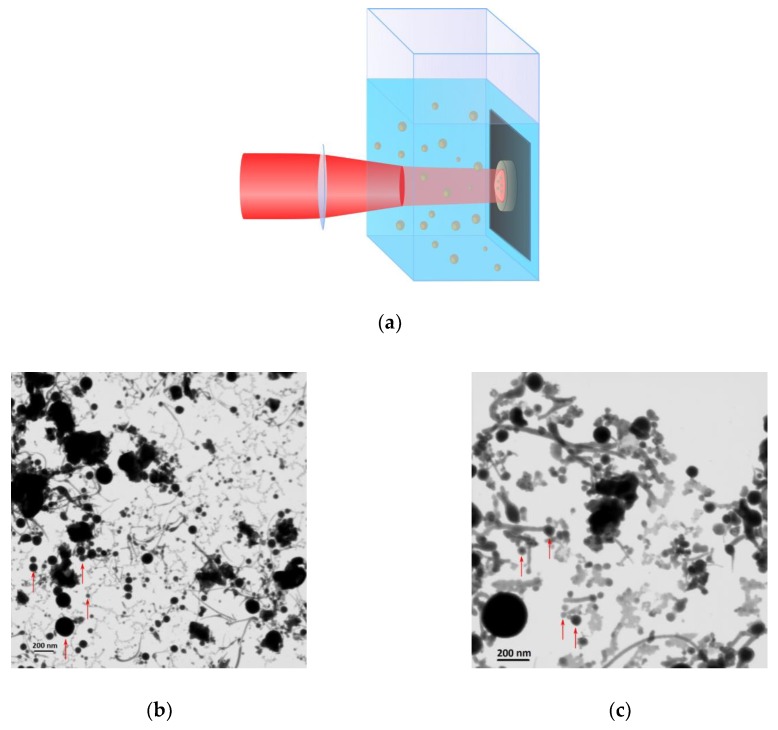
Laser ablation setup and results. (**a**) Schematic representation of the ablation geometry. Typical transmission electron microscopy (TEM) images of nanoparticles (NPs) obtained by laser ablation of the Sm oxide target with (**b**) 10 µJ and (**c**) 100 µJ pulse energies.

NPs were synthesized by ultra-shot (fs) laser ablation of the Sm oxide target in deionized water under ambient conditions. Schematics of the ablation geometry are drawn in Figure 2a. Briefly, the Sm oxide target was fixed vertically on the wall of a BK-7 glass vessel filled with 14 mL of ultrapure water (18.2 MΩ cm at 25 °C). A 3 mm diameter beam from a Yb:KGW laser (1030 nm wavelength, 270 fs pulse duration, up to 400 µJ pulse energy, 1–100 kHz repetition rate, TETA 10 model, Avesta, Moscow, Russia) was focused by a 75 mm lens on the surface of the target, through a side wall of the vessel. The thickness of the liquid layer from the entrance glass to the target surface was 17 mm. The ablation was performed with different laser pulse energies ranging from 10 up to 100 µJ. The interaction of powerful fs laser pulses with water was accompanied by energy-dependent self-focusing of the laser beam [31]. Indeed, the power of laser pulses used in our experiments was in the range of 37 to 370 MW, which significantly exceeds the self-focusing threshold of water at 1030 nm (14.8 MW [32]). Therefore, to take into account shifts of the focal plane due to the energy-dependent self-focusing effect, the position of the focusing lens was adjusted to keep optimal focusing conditions, which we determined as yielding the maximum productivity of the ablation process, measured by weighing the target before and after ablation. The duration of each experiment was 1 h. The ablation vessel was mounted on a platform which performed continuous scanning over a 2 × 2 mm area with the 5 mm/s speed in order to avoid ablation from the same area. The ablation target and the ablation chamber were cleaned each time before and after an ablation experiment by using an ultrasonication step in analytical-grade acetone, followed by ultrasonication in deionized water and finally thorough rinsing in ultrapure water.

**Figure 3:**
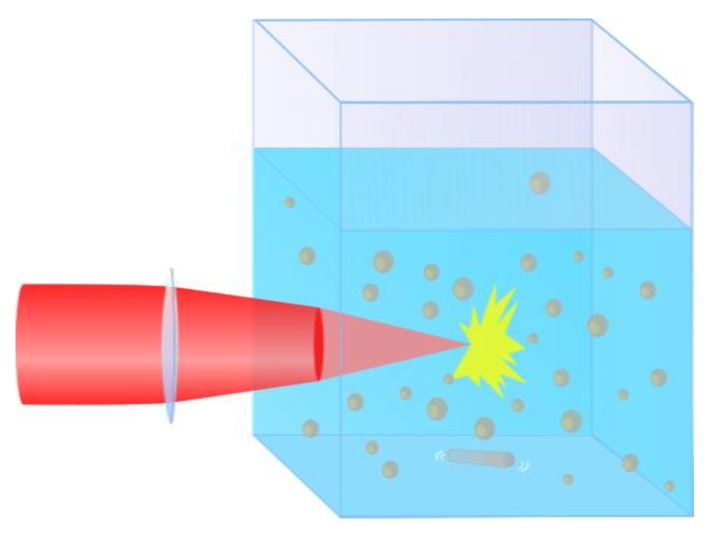
Schematic representation of the fragmentation geometry.

As an additional control over the NPs size, we applied the technique of fs laser fragmentation which was developed in our previous works. A schematic of the fragmentation geometry is drawn in Figure 3. Briefly, a solution of Sm oxide NPs first prepared by the laser ablation step, was illuminated by the same laser in the absence of the target. The same optical scheme from the laser ablation setup was used, but the laser beam was focused into the solution 1 cm behind the entrance glass rather than on the target surface. The solution was continuously homogenized by a magnetic stirrer during the fragmentation process. The used fragmentation geometry allowed illumination only of a small fraction of the liquid volume at any given moment; therefore, a prolonged processing time (5 h) was required to fragment most of the particles in the solution.

### 2.3. Characterization of Nanoparticles

Morphology, structure, size and composition of the synthesized NPs were characterized by a scanning transmission electron microscopy (STEM) system (MAIA 3, Tescan, Czech Republic) operating at 0.1–30 kV coupled with an EDS detector (X-act, Oxford Instruments, High Wycombe, UK). Samples for electron microscopy were prepared by dropping 1 μL of the NPs solution onto a carbon-coated copper grid (for transmission electron microscopy (TEM) imaging) or a cleaned silicon substrate (for scanning electron microscopy (SEM) imaging) and with subsequent drying at ambient conditions. ζ–potential measurements were performed using a Zetasizer ZS instrument (Malvern Instruments, Orsay, Paris, France). The concentration of each NPs solution was determined by measuring the target weight before and after the ablation step and dividing this mass difference by the ablation liquid volume.

## 3. Results

Colloidal solutions of bare Sm oxide NPs were prepared by two-step fs laser ablation (see Materials and Methods section) of a bulk Sm oxide target in water, similar to the procedure reported in our previous works [18,20]. The ablation was performed with different pulse energies ranging from 10 up to 100 µJ, which resulted in NPs solutions of 30–50 µg/mL concentrations. In all cases, the obtained solutions were almost completely transparent, with a slight whitish coloration. The ablation geometry and typical TEM images of Sm oxide NPs synthesized with different pulse energies are shown in Figure 2. Surprisingly, the morphologies of Sm oxide NPs were very diverse, one can distinguish four fractions: (i) Relatively large randomly shaped particles with sizes from 100 nm up to 1 µm, (ii) thread-like structures with 20–40 nm thickness and 200–500 nm length, (iii) amorphous particles with sizes up to 100 nm and, (iv) spherical NPs with sizes from 10 to 100 nm (marked by red arrows in Figure 2b,c).

**Figure 4:**
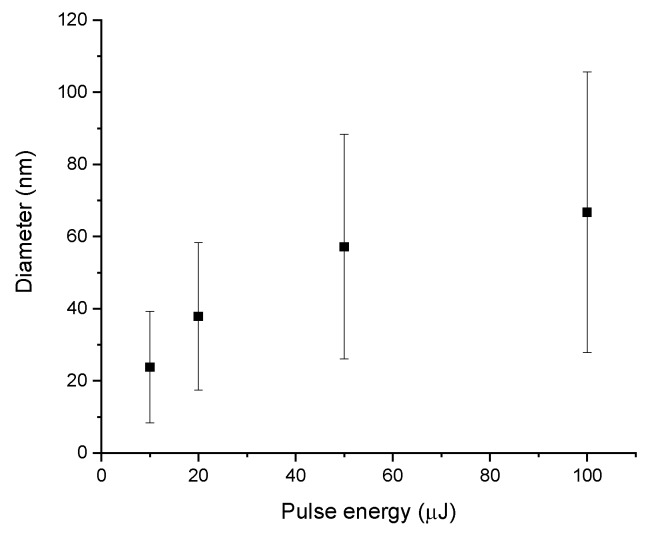
Diameter of spherical Sm oxide NPs obtained by laser ablation at different pulse energies. Data points and scale bars represent mean size and standard deviations from lognormal fit of number-weighted size hystograms.

First three fractions did not demonstrate any noticeable dependence of their morphological properties on the pulse energy. This observation can be explained by a non-laser-ablative origin of such NPs. For example, the size of large randomly shaped particles (100 nm–1 µm) was very similar to the size of initial micro powder grains, and therefore they could be produced by mechanical detachment from the target during the ablation process. Indeed, the problem of mixing of grains from a pressed powder-based target with truly laser-ablated particles has been reported by many authors [33,34]. The reason for such a mechanical ablation is the collapse of a cavitation bubble, which emerges at the liquid–target boundary, promptly after a laser pulse due to fast liquid heating by the ablated material. In the case of fs ablation, it is possible to completely suppress the cavitation bubble and the related mechanism of ablation, by the decrease of laser energy [16]; however, it leads to very low ablation productivity. At the same time, the formation of thread-like and amorphous particles (fractions (ii) and (iii)) could be explained by thermal and oxidative mechanisms accordingly. In contrast, spherical NPs tended to increase their size, with an increase of the laser pulse energy, as is shown in Figure 4 (size histograms for each data point from Figure 4 are shown in Figure S1). The mean size of the spherical NPs increased from 20 nm to 70 nm, when the laser ablation energy was increased from 10 to 100 µJ. This observation is consistent with the dependence of the NPs size on pulse energy during laser ablation for other materials.

We observed some sedimentation of particles during the first 24 h after the laser-ablative preparation of solutions. This sedimentation can be attributed to precipitation of the large size mechanically-ablated particles, as discussed previously. After that, the remaining solutions were stable and did not show any traces of precipitation during their storage under room conditions for several months. Such a good colloidal stability of bare laser-synthesized colloids is dictated by electrical charging of NPs during the ablation process and related electrostatic stabilization. Indeed, according to our ζ—potential measurements, the surface potential of the laser-ablated Sm oxide NPs was +20 mV which coincides with the stability threshold for colloidal solutions.

**Figure 5:**
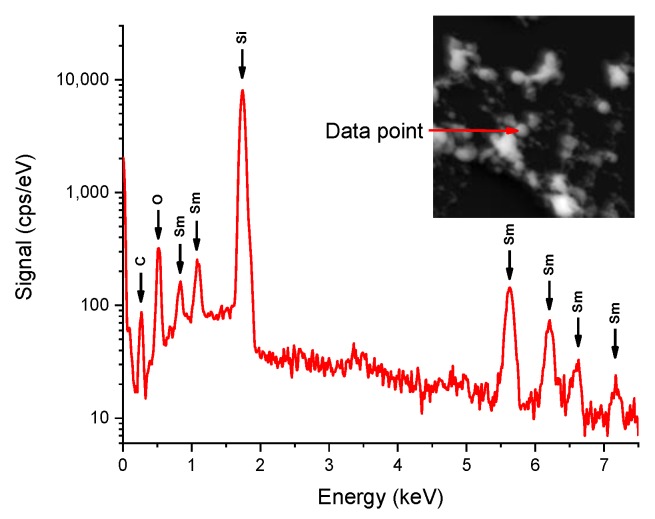
Energy-dispersive X-ray spectroscopy (EDS) spectrum of synthesized NPs. Strong signals from samarium and oxygen are related to synthesized NPs. The silicon peak is related to the Si substrate, while the carbon signal is related to organic contamination of the vacuum chamber.

The composition of synthesized NPs was qualitatively analyzed by energy-dispersive X-ray spectroscopy (EDS). A typical EDS spectrum is shown in Figure 5. As one can see from the figure, NPs were mainly composed of samarium and oxygen. Unfortunately, this experimental technique does not allow one to precisely measure the stoichiometry of NPs. Therefore, we could only determine the presence of elements, but not their ratio. The large silicon (Si) peak in the spectrum is explained by the use of the Si wafer as a substrate for the measurement, while the carbon (C) signal comes from a common organic contamination of the measuring vacuum chamber. While the EDS technique does not show the presence of hydrogen, the formation of Sm hydroxide along with Sm oxide NPs is also possible as reported in ref. [9]. Note that the chemical composition of NPs synthesized by laser ablation does not always reproduce the composition of the ablation target, while the composition of NPs generally depends on the chemical stability of the ablation target material in the solvent. However, since the therapeutic efficacy of our NPs for intended biomedical application is dependent only on the amount of ^152^Sm isotopes in NPs, a very high enrichment degree (97.78–98.70%) of our target guarantees its high concentration in the composition of NPs.

**Figure 6:**
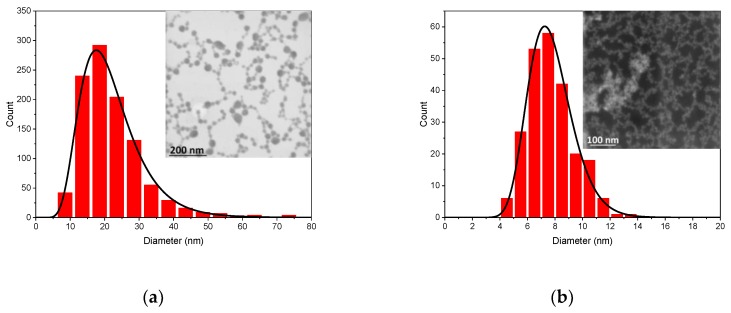
Size distributions with typical scanning transmission electron microscopy (STEM) images of Sm oxide NPs after fs LFL at: (**a**) 50 µJ and (**b**) 100 µJ pulse energies.

This one-step laser ablation cannot provide good control of size characteristics, as formed NPs have a large mean size and are size- and shape-dispersed. Such characteristics are hardly consistent with projected applications. To homogenize size distributions, we applied the technique of femtosecond laser fragmentation in liquids (LFL), which was developed in our earlier works to reduce the size of Au and TiN NPs [18,23]. The technique implies irradiation of a preliminary prepared colloidal solution by a focused fs laser beam (see Materials and Methods section). This approach profits from a significant broadening of an intense fs laser spectrum and a generation of a so-called white light supercontinuum, which leads to the efficient fragmentation of almost any NPs, regardless of their absorption profile. The results of such an additional laser treatment are shown in Figure 6. As one can see, the fs LFL not only allows one to drastically decrease the size of NPs and narrow down their size distribution, but also provides a size control. Indeed, a prolonged LFL with 50 µJ pulse energy led to a log-normal size distribution of Sm oxide NPs with 20 nm mean diameter and 10 nm FWHM width, while LFL with 100 µJ pulse energy resulted in NPs with 7 nm mean diameter. This result is consistent with our previous results on fs laser fragmentation of Au NPs [18]. Here, under certain conditions, we could also observe the decrease in NPs size under the increase of laser fluence. It is important to note that after the fs laser fragmentation step, colloidal solutions did not show any sign of precipitation, which was obviously due to homogenation of the NPs sizes.

Thus, by combining the femtosecond laser ablation and fragmentation steps we managed to fabricate low size-dispersed samarium oxide NPs with their mean size depending on laser fluence.

## 4. Discussion

### 4.1. Laser Ablation

**Figure 7:**
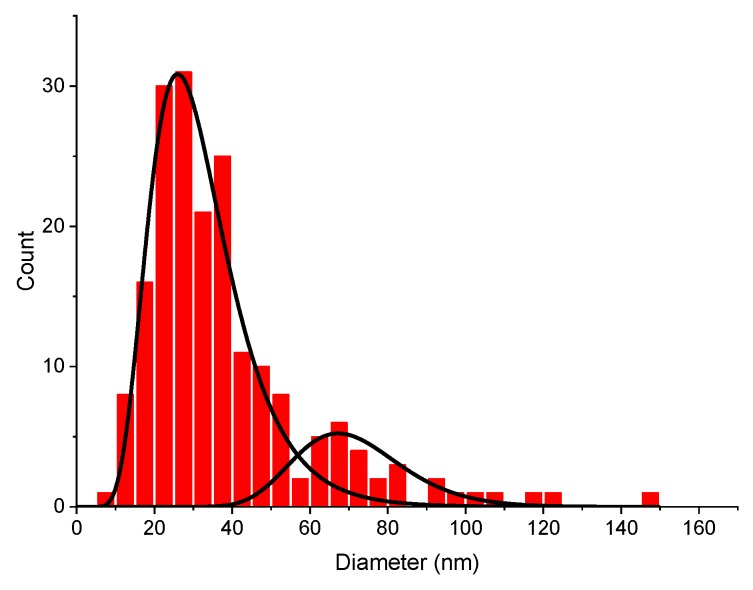
Bimodal number-weighted size distribution of spherical Sm oxide NPs fraction obtained by laser ablation. “Primary” NPs are centered around 25 nm, while “secondary” around 65 nm.

Physicochemical processes involved in the formation of NPs during PLAL are very complex and most of them possess a transient character. Therefore, an exact understanding of where, when and how NPs form and grow is still lacking; however, there are a number of widely accepted theoretical and experimental findings which address these questions. Femtosecond laser ablation at low laser fluences generally leads to ejection of atoms and nanoscale clusters [35,36], while their amount depends on the laser fluence. These ablated atoms and clusters could then form NPs in a confined region near the target surface, via a dynamic formation mechanism proposed by Mafune et al. [37], with an increase of the NPs mean size resulting from the increase of laser fluence. The same tendency was observed in this study for spherical Sm oxide NPs as shown in Figure 4. However, when the laser fluence is high enough, additional factors related to the high energies of ejected species forming a hot plasma near the ablation spot must be taken into account. The plasma can become intense enough to produce an additional thermal mechanism of material ablation [16,38], resulting in melting and evaporation of the target material and, as a consequence, the ejection of much larger NPs, and the formation of filaments and needle-like structures occur. Moreover, energy exchange of plasma with the surrounding liquid leads to the formation of so-called cavitation bubbles [39,40]. The collapse of this bubble is accompanied by a significant release of mechanical energy [40], which could cause secondary ablation of the material [38]. We believe that the collapse of the cavitation bubble led to a mechanical decomposition of the near-ablation-spot region of the relatively fragile target, formed by pressing a Sm oxide micro powder. As a result, a significant amount of large (>200 nm) randomly shaped particles was found in TEM images of ablated samples (Figure 2b,c). The presence of sub-micrometer particles in colloidal solutions is detrimental for a number of reasons: (i) Such particle are not suitable for projected biomedical and catalytic applications, because they are either less effective in passive tumor targeting or they have a lower specific surface compared to sub 100 nm particles; (ii) the large mass of such particles significantly distorts NPs concentration measurements; (iii) the presence of >200 nm particles practically limits colloidal stability, since it is almost impossible to electrostatically stabilize such heavy particles against precipitation. Another consequence of plasma-induced thermal ablation and the cavitation bubble-related effects is in the appearance of a bimodal size distribution (Figure 7 and Figure S1) of the spherical fraction of laser-ablated NPs.

We believe that the presence of small (often called “primary”) NPs (~25 nm in this case) can be attributed to the pure radiation-based ablation of nanoclusters and their subsequent coalescence in the water environment, while the presence of the large (often called “secondary”) NPs (~65 nm in this case) can be related to plasma and cavitation bubble effects [41,42].

### 4.2. Laser Fragmentation

Two mechanisms of LFL are typically considered: (i) Photothermal evaporation [43] and (ii) Coulomb explosion [44,45]. The size reduction due to the first mechanism is achieved via heating of particles above the melting point (usually heating up to the boiling point is considered) and subsequent evaporation of the particles’ material. Such a thermal mechanism is more relevant for long (ns) laser pulses [46]. In the second model, electrons, highly excited by laser radiation, are ejected leaving positively charged NPs which immediately rupture due to an internal charge repulsion. This regime requires high temperatures of the electronic subsystem, which are easier to achieve during the interaction of short laser pulses (sub ns) with matter. Therefore, the Coulomb explosion mechanism is more relevant for fs LFL [46].

Laser fragmentation usually results in NPs of a certain size. The final value of NPs size depends on the laser parameters and particles properties [18,46]. Such size selectivity is achieved due to the fact that the absorption cross-section of the product NPs is smaller than that of the initial particles [47]. Heat transfer from NPs to the surrounding liquid induces formation of vapor bubbles around them [48]. It was shown that the threshold of formation of such vapor bubbles is close to the NPs fragmentation threshold and exhibit a “bathtub” profile as a function of the NPs diameter [49]. Therefore, more energy per pulse is required to produce smaller NPs by LFL. These theoretical findings are consistent with the experimental results presented in this work. Indeed, as shown in Figure 6a,b a higher energy is required in order to produce smaller samarium oxide NPs.

Moreover, LFL with 100 µJ pulse energy resulted in NPs with a mean size of 7 nm, which is a very important result from the point of view of biomedical applications of such NPs. This is due to the fact that in the human organism, the renal glomerular filtration size range is about 8 nm [50,51]; therefore, everything which is smaller can quickly be removed from an organism through the kidney and so the problem of nanomaterial accumulation in organs can easily be avoided.

## 5. Conclusions

We elaborated the technique of femtosecond laser ablation and fragmentation to fabricate isotope-enriched samarium oxide nanoparticles. Whereas laser ablation from micropowder-pressed target led to a wide dispersion in size and shape of the formed NPs, a good control of NPs size parameters could be achieved by applying an additional fragmentation step. The final product presented NPs with a narrow size dispersion and variable mean size. The formed NPs are of interest for biomedical and catalysis applications. In the next phase of our study, we plan to use these nanoparticles as beta emitters in nuclear therapy.

## Supplementary Materials

The following are available online at https://www.mdpi.com/2079-4991/10/1/69/s1, Figure S1: Size histograms of spherical fraction of Sm oxide NPs, obtained by laser ablation at (a) 10 µJ, (b) 20 µJ, (c) 50 µJ and (d) 100 µJ laser energies. The second mode of large NPs could be seen on size distribution at (a) 50 nm, (b) 65 nm, (c) 145 nm and (d) 210 nm..
