# Supplementary material for: Laser-Ablative Synthesis of Isotope-Enriched Samarium Oxide Nanoparticles for Nuclear Nanomedicine
Source: Nanomaterials (Basel). 2019 Dec 28;10(1):69. doi: 10.3390/nano10010069 (PMC7022655; doi:10.3390/nano10010069)
Supplement: Supplementary file 1 [file nanomaterials-10-00069-s001.zip › nanomaterials-655905-SI.pdf]

Supplementary Materials: Laser-Ablative Synthesis of Isotope-Enriched Samarium Oxide Nanoparticles for Nuclear Nanomedicine

Elena Popova-Kuznetsova [1], Gleb Tikhonowski [1], Anton A. Popov [1,*], Vladimir Duflot [2], Sergey Deyev [1,3], Sergey Klimentov [1], Irina Zavestovskaya [1,4], Paras N. Prasad [1,5,*] and Andrei V. Kabashin [1,6,*]

[1] Bionanophotonic Lab., Institute of Engineering Physics for Biomedicine (PhysBio), National Nuclear Research University MEPHI, Moscow 115409, Russia; EAPopovaKuznetsova@mephi.ru (E.P.-K.); gtikhonowski@gmail.com (G.T.); SMKlimentov@mephi.ru (S.K.); INZavestovskaya@mephi.ru (I.Z.)
[2] Karpov Institute of Physical Chemistry, NIFKhI, Obninsk 249033, Kaluga region, Russia; duflot@mail.ru
[3] Shemyakin–Ovchinnikov Institute of Bioorganic Chemistry, Russian Academy of Sciences, Moscow 117997, Russia; deyev@ibch.ru (S.D.)
[4] Lebedev Physical Institute of the Russian Academy Sciences, Moscow 119991, Russia
[5] Department of Chemistry and Institute for Lasers, Photonics, and Biophotonics, University at Buffalo, The State University of New York, Buffalo, NY 14260, USA; pnprasad@buffalo.edu (P.N.P.)
[6] LP3, Aix Marseille University, CNRS, Marseille 13288, France; kabashin@lp3.univ-mrs.fr (A.V.K.)
* Correspondence: AAPopov@mephi.ru (A.A.P.); pnprasad@buffalo.edu (P.N.P.); kabashin@lp3.univ-mrs.fr (A.V.K.)

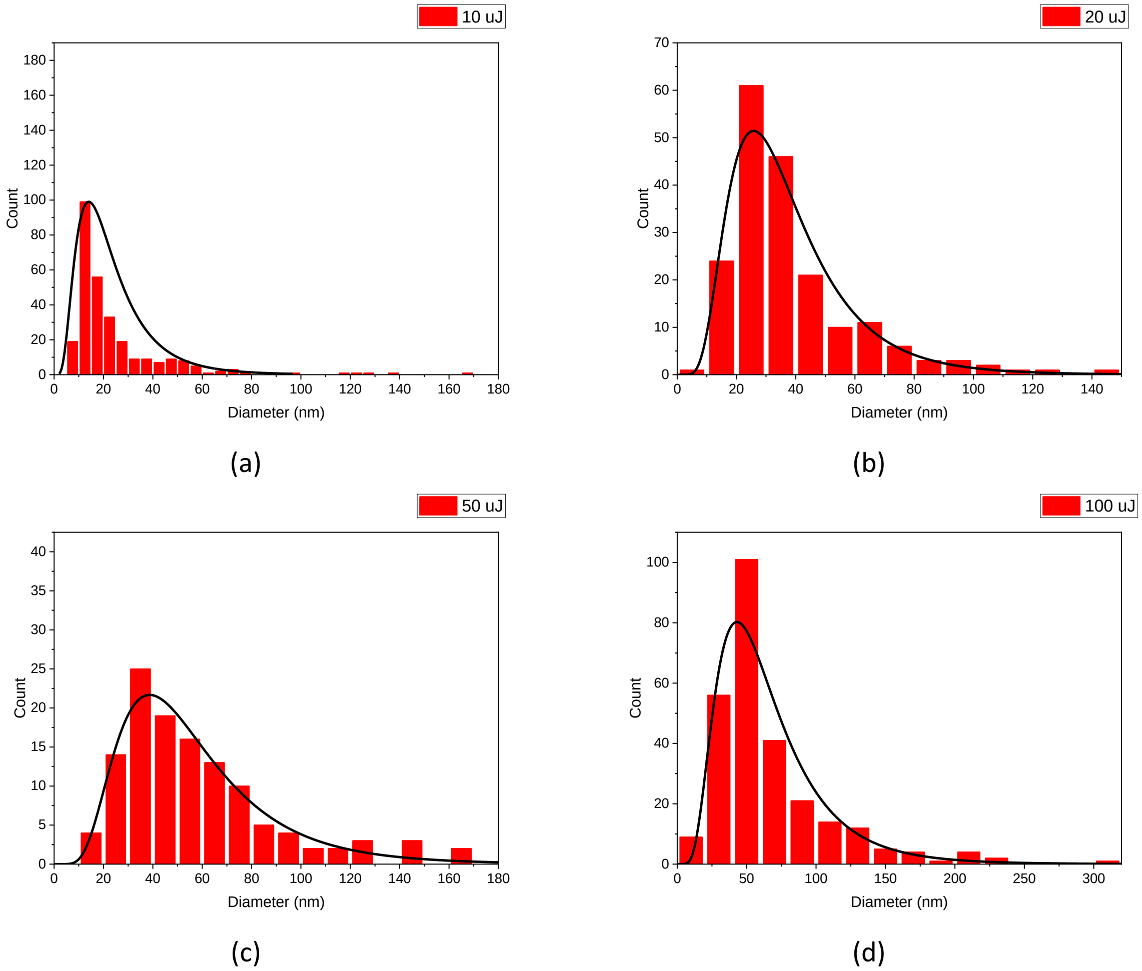

Figure S1. Size histograms of spherical fraction of Sm oxide NPs, obtained by laser ablation at (**a**) 10 μJ, (**b**) 20 μJ, (**c**) 50 μJ and (**d**) 100 μJ laser energies. The second mode of large NPs could be seen on size distribution at (**a**) 50 nm, (**b**) 65 nm, (**c**) 145 nm and (**d**) 210 nm.
